# Mapping of IgE-binding regions on recombinant Cyn d 1, a major allergen from Bermuda Grass Pollen (BGP)

**DOI:** 10.1186/1476-7961-7-3

**Published:** 2009-02-02

**Authors:** Ruby Tiwari, Prem L Bhalla, Mohan B Singh

**Affiliations:** 1 ARC Centre of Excellence for Integrative Legume Research, Faculty of Land and Food Resources, University of Melbourne, Parkville, Australia; 2 Institute of Food Nutrition and Human Health, Massey University, Palmerston North, New Zealand

## Abstract

**Background:**

Bermuda grass (*Cynodon dactylon*; subfamily Chloridoideae) is an important source of seasonal aeroallergens in warm tropical and sub-tropical areas worldwide. Improved approaches to diagnosis and therapy of allergic diseases require a thorough understanding of the structure and epitopes on the allergen molecule that are crucial for the antigen-antibody interaction. This study describes the localization of the human IgE-binding regions of the major group 1 pollen allergen Cyn d 1 from Bermuda grass.

**Methods:**

A cDNA library was constructed from Bermuda grass pollen (BGP) using a Lambda gt11 expression vector. The gene encoding the Cyn d 1 allergen was isolated by screening the library with a mouse monoclonal antibody raised against grass group 1 allergen. In order to characterize the IgE epitopes on Cyn d 1, seven overlapping fragments and three deletion mutants were cloned and over-expressed in E. coli. The recombinant fragments and deletion mutants were evaluated for their comparative IgE reactivity with sera of non atopic individuals and grass pollen allergic patients by ELISA and a dot-blot assay.

**Results:**

Analysis of IgE binding regions by overlapping fragments and deletion mutants identified two major allergenic regions corresponding to amino acids 120–170 and 224–244. Deletion of either or both regions led to a significant reduction in IgE binding, emphasizing the importance of the C-terminal region on Cyn d 1 in epitope-IgE interaction.

**Conclusion:**

Anti-Cyn d 1 IgE antibodies from allergic human sera recognize two epitopes located at the C-terminal end of the molecule. These data will enable the design of improved diagnostic and therapeutic approaches for BGP hypersensitivity.

## Background

It is estimated that up to 20% of the population in developed countries suffers from atopic diseases caused by airborne allergens derived from grass and tree pollen, house dust mites and animal dander, which has a significant impact on quality of life and economic consequences [1,2]. The role of grass pollen allergens in triggering immunoglobulin E (IgE)-mediated type I allergic diseases, such as allergic rhinitis, conjunctivitis, and bronchial asthma is very well established [3,4]. Epidemiologic studies have revealed that allergies to Bermuda grass (*Cynodon dactylon*; subfamily Chloridoideae) mainly affect people in warm tropical and sub-tropical areas of the world, such as southwestern United States, South Africa and northern and central Australia. Furthermore, approximately 27% of asthmatic patients in Taiwan are reported to have a hypersensitive response to crude Bermuda grass pollen (BGP) extract [5].

Cyn d 1, the major allergen of BGP, is the most abundant protein component of BGP, comprising 15–20% of crude pollen extract. It dominates the human IgE response with 87% of individuals allergic to BGP showing positive reaction to this 32 kDa protein [6,7]. Clinical evidence of cross-reactivity among grass pollens has suggested that diagnosis and effective immunotherapy can be achieved with a limited number of grasses, although the selection of species to treat against is based on regional prevalence and taxonomic relationship. Grass group 1 allergens, with molecular weights in the range of 31–35 kDa, are recognized as one of the most prominent allergenic components of pollen extracts. In recent years, the genes encoding Group 1 allergens for several grasses have been cloned and expressed using molecular biology techniques [8,9]. When translated, the cDNA sequences predict proteins of approximately 240 amino acid residues and molecular weights of about 26 kDa. They share an N-glycosylation site at amino acid position 9 and carry glycans that account for 4–5% of their molecular weight. These glycoproteins are located in the cytoplasm of the pollen grain and are rapidly released when hydrated upon contact with moist mucosal surfaces. A characteristic feature of group 1 allergens is the presence of seven strictly conserved cysteine residues, located mainly in the N-terminus of the protein [10-12].

The first full length cDNA coding for Cyn d 1 was identified by Smith et al [11] followed by Chang et al [12]. Au and colleagues have suggested the existence of two groups of Cyn d 1, the acidic and the basic. The Cyn d 1 cDNAs belonging to the basic group have N-terminal sequences of A*I*GDKPNITATYG*SK*WL*E*, while the sequences of acidic isoallergens showed substitutions of M, D, L, D for I, S, K, E (italicised in the aforementioned sequence), respectively [13].

Comparison of amino acid sequences of group 1 allergens reveals a high degree of sequence similarity, which highlights a possible basis of allergenic cross-reactivity observed among group 1 allergens when investigated by inhibition techniques among taxonomically related grasses [14-16]. However, earlier studies on allergens from BGP have shown that BGP allergens share minimal IgE cross-reactivity with pollen from Poaceae sub-family grasses, such as *Dactylis glomerata*, *Lolium perenne*, *Festuca pratensis*, *Phleum pratense *and *Poa pratensis *when tested with crude pollen extracts. These findings suggest the presence of unique IgE epitopes in BGP allergens [17,18]. In a recent study, twenty specific anti-Phl p 1 monoclonal antibodies (MAbs) were produced from BALB/c mice immunized with natural Phl p 1. When tested for specificity with thirteen different grass pollen extracts, eighteen to nineteen anti-Phl p 1 MAbs recognized the homologous allergen in pollen extracts from grasses of the Poeae tribe while only four MAbs recognized the group 1 allergen from *Cynodon dactylon *[19]. These studies further suggest that the antigenic regions of Cyn d 1 in BGP may be different from Poeae grasses, and as a result, individuals allergic to BGP may require separate diagnosis and therapy.

Recombinant allergens are useful tools for structural analysis, including that of IgE binding epitopes, and may provide a better understanding of the allergic immune response. Although allergenic properties and IgE epitopes from Poaeae grasses have been studied extensively [20-22], information on the IgE epitopes of Cyn d 1 is still lacking. Studies of this nature are important because a sizable population is exposed and sensitized to BGP, and it seems to be immunologically distinct from other clinically important allergenic grasses. In light of the prevalence of BGP in warm tropical regions and its role as a respiratory allergen, we focussed on identification of the immunodominant regions of the major BGP allergen Cyn d 1. To locate the relevant IgE binding regions on Cyn d 1, a series of seven overlapping recombinant peptides and three deletion mutants generated from the cDNA of Cyn d 1 were investigated for their IgE binding capacity. We report the presence of at least two major IgE binding regions located within the C-terminus of Cyn d 1.

## Methods

### Pollen extract and patient sera

Human allergic sera were obtained from patients with a clinical history and positive skin test reaction to Bermuda grass pollen. Normal control serum samples were obtained from healthy non-atopic subjects with a negative skin test to BGP. BGP was obtained as dry, non-defatted pollen from Greer Laboratories (North Carolina, USA). Pollen extracellular proteins were extracted by incubation in PBS (10 mM Na_2_HPO_4_, 1.8 mM KH_2_PO_4_, 140 mM NaCl, 2.7 mM KCl, pH 7.4) for 10 min at room temperature with periodic stirring. The extract was filtered through a Whatman No. 1 filter and centrifuged at 5000 g for 30 min at 4°C to separate the supernatant containing the secreted protein. The serine protease inhibitor phenylmethylsulphonyl fluoride (PMSF [0.05% v/v]) was added to the extract to prevent protein degradation due to protease activity. Both sera and pollen were stored at -20°C until use.

### Isolation of messenger RNA from Cynodon pollen

*Cynodon dactylon *pollen was purchased from Greer Laboratory (North Carolina, USA). Total RNA was isolated from 500 mg pollen using a slightly modified guanidine hydrochloride method described by Logemann and co-workers [23]. Pollen grains were finely ground in liquid nitrogen, and mixed in 5 mL of extraction buffer (7.5 M guanidine HCl in 0.1 M β-mercaptoethanol, 0.5% w/v lauryl sarcosinate and 25 mM sodium citrate) prepared in diethyl pyrocarbonate (DEPC) treated water. The extract was centrifuged at 12,000 g for 20 min. The supernatant was collected and purified twice with equal volume of phenol: chloroform: isoamylalcohol (25:24:1 v/v) followed by centrifugation. The RNA was treated with DNase and the quality was analysed by agarose gel electrophoresis. Aliquots were stored at -70°C. Poly (A) messenger RNA was affinity purified using oligo-dT cellulose (Microfast trak Invitrogen), following the manufacturer's instructions.

### Construction and immunoscreening of the Bermuda grass pollen cDNA library

Double-stranded cDNA was synthesized from purified mRNA using a synthesis kit (Pharmacia LKB, Uppsala, Sweden) according to manufacturer's instructions. The cDNA was ligated to an Eco R1/Not 1 adaptor, cloned into a Lambda gt 11 vector (Amersham), and packaged into bacteriophages (Gigapak Gold III Stratagene) to construct a cDNA expression library. E. coli Y1090 competent cells were transduced with *in vitro *packaged phages, which resulted in a primary library with 98% recombinant clones. The cDNA library was screened with anti-Cyn d 1 mouse mAb 3A2, which binds Cyn d 1. Screening of the cDNA library was carried out by taking plaque lifts and probing with mAb 3A2. Briefly, lambda-gt11 phage with the cDNA inserts were used to infect E. coli Y1090 cells, plated onto LB plates (density 200–300 plaques/plate) containing 100 μg/mL ampicillin and incubated at 42°C for 4 h. The plates were overlaid with nitrocellulose filters (Hybond-C extra, Amersham, U.K.) soaked with 1 mM IPTG (isopropyl-β-D-thiogalacto-pyranoside) and incubated at 37°C in order to induce the expression of fusion protein. Two lifts were taken from each plate at 4-hour intervals. Subsequently, the membranes were washed with PBS-Tween20, blocked with 10% skim milk powder in PBS and incubated with mAb 3A2 (1:1000 in 1% BSA/PBS) for 5–6 hours. The membranes were washed with PBS-0.05% Tween20 and incubated with alkaline phosphatase conjugated anti-mouse IgG, 1:1000 (Sigma) at room temperature for 2 h. The positive clones were selected by colorimetric development of membranes with 0.4 mmol/L nitroblue tetrazolium (Boehringer, Mannheim, Germany), 0.4 mmol/L 5-bromo-4-chloro-3-indolyl phosphate p-toluidine salt (Astral Scientific, NSW, Australia). The plaques for positive clones were excised and further verified by secondary screening.

### Isolation of Lambda DNA of positive clones

Lambda lysates were prepared by incubating an isolated plaque with 500 μL of plating E. coli Y1090 cells for 15 min, after which the volume was made up to 5 mL with LB containing 100 μg/mL ampicillin, 5 mM calcium chloride, 0.4% w/v maltose and the culture incubated at 37°C, with shaking, for 5 h or until the bacterial cells were lysed; thereafter 500 μL of chloroform was added to each culture and incubated for a further 15 min. Bacterial debris was removed by centrifugation at 8000 g for 10 min. The lambda lysate was treated with DNase (10 μg/ml) and RNase (20 μg/ml) at 37°C for 30 minutes to remove traces of bacterial genomic DNA and RNA. Lambda DNA was isolated twice from liquid lysates with an equal volume of phenol: chloroform: isoamylalcohol (25:24:1 v/v) and the suspension gently mixed for 20 minutes followed by centrifugation at 12,000 g for 20 minutes at 4°C. Final extraction was carried out with equal volume of chloroform: isoamylalcohol (24: 1). The supernatant was transferred to a fresh tube and RNA was precipitated with 1/10 volume of 3 M sodium acetate and equal volume of cold isopropanol. The mixture was stored at -20°C and DNA pellet was recovered by centrifugation at 4°C for 20 minutes at 12,000 g. The pellet was washed with 70% ethanol, vacuum dried and dissolved in TE buffer (pH 7.4).

### Cloning and Sequencing of positive cDNA clones

The cDNA inserts of various clones were identified by PCR with lambda-gt11 forward and reverse primers on purified DNA template. The resulting PCR products were gel purified (Jetsorb Kit, Astral Scientific) and sub-cloned into pGEM-T vector (Promega). Identity of the clones was verified by automated sequencing (Applied Biosystems, USA) using T7 and SP6 DNA sequencing primers. The nucleotide sequence and deduced amino-acid sequences were compared by FASTA with those in the GeneBank database. Sequence alignments were performed with the CLUSTAL W package.

### PCR cloning of rCyn d 1 and its derived, overlapping fragments and deletion mutants

**Figure 1:**
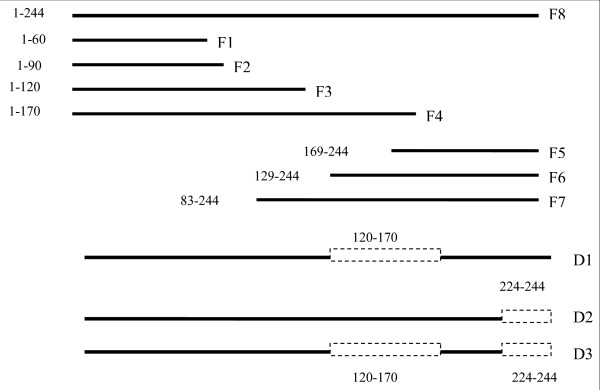
**Mapping of IgE binding regions on rCyn d 1**. Schematic location of the recombinant fragments and deletion mutants on the complete rCyn d 1 fragment. The dotted unfilled bars represent the deleted portions on the rCyn d 1 molecule.

Figure 1 shows the schematic diagram of the strategy for the generation of the cDNA fragments. The 7 overlapping fragments spanning the entire rCyn d 1 allergen sequence were amplified by PCR and restriction sites were introduced at both ends. The inserts were cloned as Stu1/Hind III-fragments in the correct reading frame downstream from the 6× His tag sequence of the pQE30Xa vector (Qiagen). The deletion mutants were cloned into the Bam H1 and Hind III restriction sites on the vector. Primer sequences for preparing the constructs are provided in Table 1. PCR was performed with 1 ng of DNA template using thirty cycles of amplifications. PCR products were analysed on agarose gels and purified using a Jetsorb kit (Astral Scientific). Purified deletion fragments were subcloned into the expression vector pQE30Xa (Qiagen) and expressed as hexa-histidine fusion proteins in E. coli M15 cells (Qiagen).

**Table 1:** Sequence of primers used for cloning rCyn d 1 and of Cyn d 1 fragments and deletion mutants in expression vector pQE30Xa.

Primer Name	Sequence (5' – 3')
λgt11Fwd	GGTGGCGACGACTCTGGAGCCCG
λgt11Rev	TTGACACCAGACCAACTGGTAATG
pQEFwd	GTGAGCGGATAACAATTTCACACAG
pQERev	GTTCTGAGGTCATTACTGGATC
F1Rev	CCATCCTT***AAGCTT***GAAGATGGGTTCGTTG
F2Rev	GATGAGGAC***AAGCTT***GGGCTCGCCGGAGC
F3Rev	CTCCTCTCC***AAGCTT***CTTCTTGGCCCATGGC
F4Rev	GCCATCGCC***AAGCTT***GGCAGCGTACTTCAC
F5Fwd	GTGAAGTAC***AGCGCT***GCCGGCGATGGCAAC
F6Fwd	CTGCGCAAG***AGCGCT***GGCGAACTGATG
F7Fwd	GCAAGGAGCCC***AGCGCT***GAGTGCTCCGGC
F8CynFwd	GGCGCATGG***AGCGCT***ATGGGCGACAAGCCG
F8CynRev	GCATCAATGC***AAGCTT***TCAGAACTGGATC
D120Fwd	CTCCTCTCC***GGATCC***CTTCTTGGCCATGGC
D170Fwd	GTGAAGTACGCT***GGATCC***GCCGGCGATGGC
D224Rev	GACATCGTCCTG***AAGCTT***TTCGACATGGCC

The sequences of the 7 overlapping fragments of varying lengths covering the entire rCyn d 1 allergen molecule (previously cloned from the cDNA expression library) were amplified using specific primers designed to introduce restriction sites at both ends and were inserted as Stu1/Hind III-fragments in the correct reading frame downstream from the 6× His tag encoding gene sequence of the pQE30Xa vector. The deletion mutants were cloned using the *Bam *H1 and *Hind *III restriction sites on the expression vector. Restriction enzyme sites are italicised and in bold.

### Expression and purification of recombinant Cyn d 1 and of Cyn d 1 fragments and deletion mutants

Briefly, a 250 mL volume of LB broth containing 50 mg/ml kanamycin and 100 mg/mL ampicillin (Sigma Aldrich) was seeded with 5 ml overnight culture of E. coli M15 cells (Qiagen) harbouring the expression plasmid with either the entire Cyn d 1 cDNA, or the seven overlapping fragments and three deletion mutants. The cultures were grown at 37°C to log phase with an optical density of A_600 _= 0.6. Isopropyl-βD-thiogalactoside (IPTG) (Progen) was added to a final concentration of 1 mM to induce expression of the fusion protein. Following incubation at 37°C for a further 3 hours, bacterial cells were harvested by centrifugation at 10,000 *g *for 20 minutes. The cell pellet from 1 mL of culture was resuspended in 100 uL of reducing SDS sample buffer and subjected to SDS-PAGE to evaluate expression levels. Cells were harvested from the culture samples by centrifugation and stored at -20°C. The cells were lysed by repeated freeze thaw cycles in liquid nitrogen. The partially lysed cells were then resuspended in lysis buffer (50 mM NaH_2_PO_4_, 300 mM NaCl, 10 mM imidazole pH 8.0). Lysozyme (Sigma, Aldrich) at 1 mg/ml was added to the resuspension, which was incubated on ice for 30 minutes for further cell lysis. RNase A (Sigma, Aldrich) at 10 μg/ml and DNase (Sigma, Aldrich) at 5 μg/ml was added to the lysate and incubated for 15 minutes to remove bacterial RNA and DNA and to reduce the viscosity of the cell lysate. The lysate was then centrifuged at 10,000 *g *for 20 min at 4°C. The supernatant was collected and added to 1 mL TALON Ni-NTA resin (Clontech, USA) to allow the resin to bind to the His tagged recombinant protein. The mixture was rocked gently on ice for 1 hour. The resin and supernatant mixture was pipetted into a 1 mL column, (Qiagen) and the column was allowed to settle by gravity before washing with a further two column volumes of wash buffer (50 mM NaH_2_PO_4_, 300 mM NaCl, 10 mM imidazole, pH 8.0). Fusion proteins were eluted from the column with several aliquots of 0.5 mL of elution buffer (50 mM NaH_2_PO_4_, 300 mM NaCl, 250 mM imidazole, pH 8.0). The purity and yield of the fusion proteins were established on 12.5% Coomassie stained SDS-PAGE gels. Concentrations were measured at OD_280_.

### Human IgE Dot blot analysis

To test the specific IgE-binding capacity of the recombinant peptides and deletion mutants, an immuno dot-blot assay was performed. Varying concentrations (300, 400 and 500 nM) of purified recombinant proteins were applied to a polyvinyl difluoride (PVDF) membrane with a Minifold I Apparatus through vacuum suction. The dot-blots were blocked with PBS (pH 7.4) containing 10% skim milk for 1 h to block free binding sites. Both pooled and individual allergic sera were diluted to 1:5 in PBS and incubated with the dot-blots overnight at 4°C. Incubation of the membranes with the secondary antibody was performed with a 1:1000 dilution of alkaline phosphatase conjugated mouse anti-human IgE (Sigma) for 1 h. The bound human IgE was detected by incubating with 0.4 mmol/L nitroblue tetrazolium (Boehringer, Mannheim, Germany), 0.4 mmol/L 5-bromo-4-chloro-3-indolyl phosphate p-toluidine salt (Astral Scientific, NSW, Australia) colorimetric substrates, and allowing the color signal on the membranes to develop. The membranes were extensively washed with PBS containing 0.1% Tween 20 after each step throughout the protocol.

### Direct ELISA of deletion mutants

The relative binding of IgE to deletion mutants and full-length rCyn d 1 was assessed in an ELISA assay. Briefly, microtiter 96-well plates (Maxisorp, Nunc, Denmark) were coated with 5 ug/well of rCyn d1 and deletion mutant proteins diluted in 0.1 M sodium bicarbonate coating buffer (pH 9.6) for O/N incubation at 4°C. Optimum concentrations of allergen and antibody were determined by checkerboard titration. Non-specific binding sites were blocked by incubating with 200 μl per well of blocking buffer (PBS, 0.05% Tween20 and 1% BSA) for 1 h at room temperature. After five washes with PBS containing 0.05% Tween20, pooled and individual sera from BGP allergic or non-allergic individuals (1:5) were added to each well and incubated O/N at 4°C. The alkaline phosphatase conjugated secondary antibody, mouse anti-human IgE (Sigma), diluted 1:1000 in PBS/0.05% Tween 20/, 1% BSA was added to the wells and incubated for 3 h at room temperature. The bound human IgE on the wells was detected by addition of 0.1% pNPP *p*-nitro-phenyl phosphate (Sigma) colorimetric substrate. The optical density was measured at 405 nm. PBS buffer and sera from non-allergic subjects were included in the assay as negative controls.

## Results

### cDNA sequence analysis of Cyn d 1

**Figure 2:**
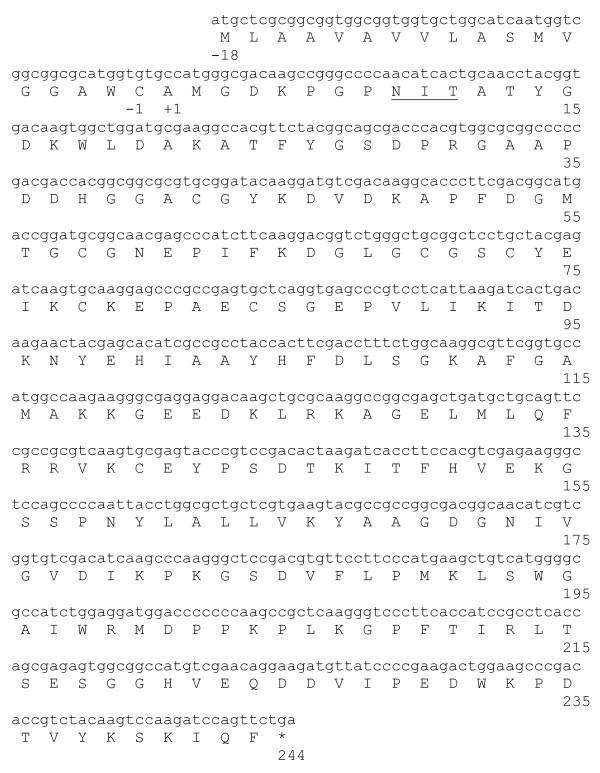
**Nucleotide sequence of Cyn d 1 and its deduced amino acid sequence**. The amino acid residues are numbered on the right. The amino acid numbering starts at the N-terminal amino acid, alanine of Cyn d 1. The putative pre-peptide (signal peptide) consists of amino acid residues -18 to -1. The putative N-glycosylation site is underlined. Asterisk indicates the stop codon.

A cDNA expression library was constructed from mRNA of BGP in λ gt11 vector. E. Coli Y1090 was transduced with in vitro packaged phages, which resulted in a plaque forming unit per millilitre of 2.2 × 10^6 ^in the primary library containing 98% recombinant clones. The library was plated at a density of 50,000 plaque forming units per 20-cm plate. A population of approximately 350,000 recombinant plaques were screened with anti-Cyn d 1 mouse monoclonal antibody mAb3A2, which selectively binds to group 1 grass pollen allergens. A cDNA encoding the full length Cyn d 1 gene with an open reading frame (ORF) of 786 base pairs (bp) starting with an ATG initiation codon and terminating with a TGA stop codon was isolated and its sequence is shown in Figure 2. The Open Reading Frame encoded a mature protein of 244 amino acids. A homology search of the nucleotide database revealed a 100% nucleotide sequence identity of Cyn d 1 cDNA with a clone in the genbank database (GenBank Database accession number AF177379) reported by Au and colleagues as an acidic isoallergen of Cyn d 1 gene [13]. This was followed by 96% sequence identity with Cyn d 1 encoding clones AF177378 and AF177380 and 93% identity with clones AF159703 and AF177030. The clone isolated from our library had the N-terminal amino acid sequence AMGDKP and was likely an acidic isoallergen with a deduced molecular weight of 27 kDa according to the classification by Au and colleagues [13]. The sequence of residues -18 to -1, which leads to the N-terminal, is composed of hydrophobic amino acids and represents the signal peptide. The motif Asn-X-Ser/Thr (underlined) at amino acid residues +9 to +11 is a putative glycosylation site. This full-length Cyn d 1 clone was used as the template for expressing recombinant Cyn d 1 protein and its derivatives for this study.

### Expression and purification of rCyn d 1 and its derived fragments and deletion mutants

**Figure 3:**
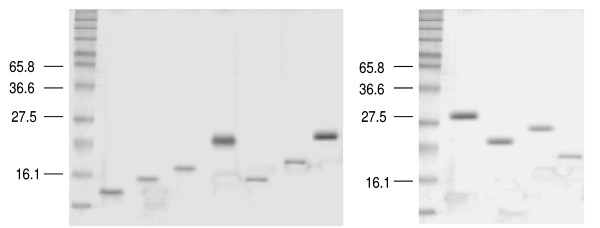
**SDS PAGE of overlapping fragmets FI-F7, rCyn d 1 (F8) and deletion mutants D1–D3**. Recombinant proteins of the different fragments were expressed in M15 cells. The purified proteins were subjected to SDS PAGE and Coomassie staining. The arrows point to purified recombinant protein bands.

We derived a full-length Cyn d 1 fragment as a cDNA clone by immunoscreening of a BGP lambda gt11 expression library with anti-Cyn d1 mouse mAb. The full-length Cyn d 1 cDNA and the fragments were cloned into a pQE30Xa expression vector and expressed as His-tagged fusion protein in M15 cells. The expression of the recombinant fragments was determined by subjecting the bacterial lysates to SDS PAGE followed by Coomassie Brilliant blue staining. The expressed and purified recombinant proteins showed bands with expected molecular mass as shown in Figure 3. The length and location of the seven overlapping fragments were F1 = 1 to 60, F2 = 1 to 90, F3 = 1 to 120, F4 = 1 to 170, F5 = 169 to 244, F6 = 129 to 244 and F7 = 83 to 244. The deduced molecular masses for these recombinant peptides F1 to F7 were 6.1, 9.3,13, 19, 9, 13 and 19 kDa, respectively. Fragment 8, which is the full-length rCyn d 1 was 244 amino acids long and had a deduced molecular weight of 27 kDa. The expressed proteins were affinity purified using a Ni-NTA resin column. Three deletion mutants (D1, D2 and D3), missing parts of the Cyn d 1 molecule, were prepared by PCR amplification, expressed as His-tagged fusion proteins and affinity purified. The overlapping fragments were designed so as to cover the entire Cyn d 1 sequence with intervals of 30 to 50 residues. The seven overlapping fragments were expressed and the crude bacterial lysates were subjected to dot blot analysis to test for IgE binding. From the preliminary dot blot assays, the additional 50 amino acid residues in F4 from F3 made it IgE reactive and therefore this stretch of 50 residues appeared to be a potential epitope-bearing region (data not shown). The C-terminal fragment F5, consisting of only 74 residues, had the ability to bind to IgE, indicating that residues 169–244 were important for IgE reactivity. Furthermore, a study by Esch and Klapper mapped an IgE binding site to the last 25 amino acids at the C-terminal end of Lol p 1, a major group 1 allergen from Rye grass pollen with high sequence homology to Cyn d 1 [24]. Based on the findings from the preliminary dot blot assays and the study by Esch and Klapper on Lol p 1, three deletion mutants D1, D2 and D3 were generated. A stretch of fifty amino acids, positioned 120 to 170, was removed in D1 since these additional residues in F4 from F3 made it IgE reactive. The last twenty residues (224–244) were removed to generate D2 based on the IgE binding mapped to the last 25 residues of Lol p 1 by Esch and Klapper [24] and a stretch of nucleotide sequence where a restriction enzyme site could be easily introduced. The third deletion mutant, D3, was a combination of D1 and D2, lacking both 50 residues from 120 to 170 as well as the last 20 residues from 224 to 244. The Cyn d 1 deletion mutants showed molecular sizes of 20.9 kDa, 24.4 kDa and 18.5 kDa respectively as shown in Figure 3. To further compare the IgE reactivity of the peptide fragments and deletion mutants, the purified proteins were subjected to IgE dot blot analysis and direct ELISA.

### Dot blot analysis of Cyn d 1 fragments and deletion mutants

**Figure 4:**
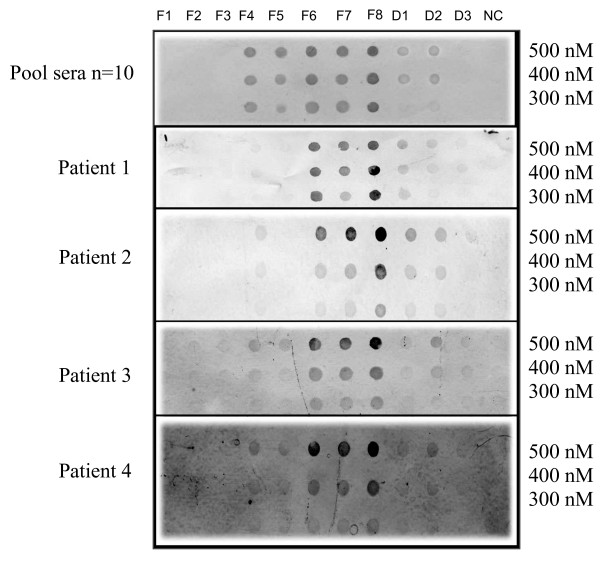
**Dot blot immunoscreening of the seven overlapping fragments (F1–F7), whole rCynd 1 and deletion mutants**. Dot blot immunoscreening of the seven overlapping fragments (F1–F7), whole rCynd 1 (F8) and deletion mutants (D1, D2, D3) with pooled sera from 10 patients and four individual patients with BGP allergy. Purified recombinant fusion proteins were applied onto PVDF membrane at 3 different concentrations (300 nM, 400 nM and 500 nM) and PBS buffer as negative control (NC).

To evaluate the IgE-reactivity of purified whole rCyn d 1 (F8), overlapping peptide fragments (F1-7) and deletion mutants (D1-3), the purified proteins were transferred onto PVDF membranes and probed with four individual and a pool of sera from 10 BGP allergic individuals. Three concentrations (300, 400 and 500 nM) of the recombinant proteins were dotted onto the membrane. Purified rCyn d 1 (F8) was used as the positive control, while PBS buffer served as negative control. The dot blots comparing the IgE binding of the recombinant fragments and deletion mutants is shown in Figure 4. All of the sera from BGP positive patients recognized intact rCyn d 1. IgE in the sera from non-allergic individuals did not bind to rCyn d 1 (data not shown). Peptide fragments F1 (1–60), F2 (1–90) and F3 (1–120) did not bind to IgE. IgE binding to fragments F4 (1–170) and F5 (169–244) were weak compared to complete rCyn d 1 fragment (F8), and the positive IgE binding was heterogeneously distributed among the different sera tested. Fragments F6 (129–244) and F7 (83-24) showed IgE reactivity almost equal and comparable to that of whole rCyn d 1 (F8). The deletion mutants D1 and D2 showed significantly weak IgE reactivity while the third deletion mutant, D3, showed almost complete depletion of IgE binding. The pattern of IgE reactivity was similar for all the sera for F6, F7 and the three deletion mutants. The immuno dot blotting results indicate that the C-terminal region harbours the major IgE binding domains (120–170 and 224–244) and is of most diagnostic relevance.

### IgE reactivity of rCyn d 1 deletion mutants by ELISA

**Figure 5:**
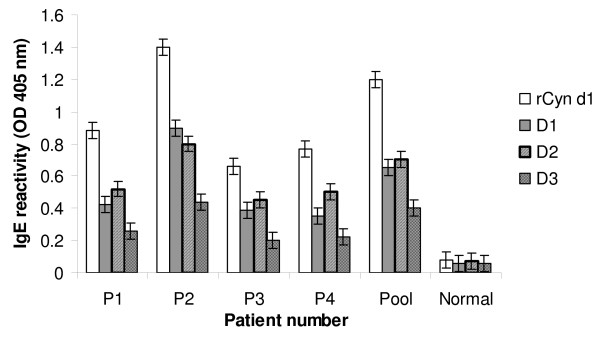
**Enzyme-linked immunosorbent assay data for serum immunoglobulin (Ig)E reactivity with whole recombinant Cyn d 1 and deletion mutants**. Enzyme-linked immunosorbent assay data for serum immunoglobulin (Ig)E reactivity with whole recombinant Cyn d 1 (unfilled bars) and deletion mutants D1 (grey bars), D2 (hatch bars) and D3 (square pattern bars) with four BGP positive individual sera (P1–P4) and allergic serum pool of 10 patients (Pool) and nonallergic serum pool of five normal subjects (Normal). OD values are expressed as mean from triplicate wells (± SEM).

A direct ELISA was performed to obtain a quantitative estimation of IgE binding of the deletion mutants. The overlapping fragments were primarily used to localize the regions on Cyn d 1 involved in IgE interaction. Once the IgE binding regions had been identified, the deletion mutants were subsequently generated and tested alongside the fragments in IgE dot blot assays. The direct ELISA was carried out on the deletion mutants to obtain a quantitative comparison of their IgE reactivity and the significance of the deleted regions from each mutant in IgE binding. Four individual sera and pooled sera from 10 grass pollen allergic patients were used to react with solid-phase bound recombinant proteins. Serum from a non-atopic donor and PBS buffer were used as negative controls. The optical density was measured at 405 nm on an ELISA plate reader, and the results are shown in Figure 5. IgE from both individual and pooled sera bound strongly to purified whole rCyn d 1. The percentage binding was calculated using the OD readings obtained through direct ELISA. The OD reading with whole purified rCyn d 1 was expressed as 100% and the OD of the deletion mutants were expressed in percentage relative to that of whole rCyn d 1. The three deletion mutants were recognized by patient IgE more weakly than the entire allergen molecule in the following order: recombinant rCyn d 1 (100%) > D2-(224–244)-(61.4 + 2.1%) > D1-(120–170)-(54.2 + 3.5%) > D3-(120–170 and 224–244)-(30.6 + 0.8%). Pooled sera and individual patient sera P1, P3 and P4 showed slightly less binding to D1 than D2 except P2, which reacted slightly more with D1 in comparison to D2. The binding profile of all sera sets indicated close to 70% reduction in IgE binding to D3, while D1 and D2 both showed about 40% reduction in reactivity in comparison to intact rCyn d 1. These data are summarized in Table 2. These results further confirmed the presence of at least two major IgE binding domains (120–170 and 224–244) on Cyn d 1.

**Table 2:** Comparison of patients' IgE binding to rCyn d 1 and deletion mutants.

**Sera**	**rCyn d 1**	**D1**	**D2**	**D3**
P1	100	47.7	59	29.5
P2	100	64.3	57	31.4
P3	100	59.1	68	30.3
P4	100	45.5	64.9	28.6
Pool n = 10	100	54.2	58.3	33.3
Mean ± SEM		54.2 ± 3.5	61.4 ± 2.1	30.6 ± 0.8

The percentages of IgE binding of deletion mutants relative to whole rCyn d 1 are shown for individual serum (P1–P4) and pooled patient sera (n = 10) as determined by ELISA. The percentage binding was calculated using the OD readings obtained through direct ELISA. The OD reading with whole purified rCyn d 1 was expressed as 100% and the OD of the deletion mutants were expressed in percentage relative to that of whole rCyn d 1. The mean ± SEM is shown in the bottom line.

## Discussion

In the present study, the cDNA of one of the most relevant environmental aeroallergens, the major Bermuda grass pollen allergen, Cyn d 1 (acidic isoform) was successfully cloned and expressed to produce immunologically active recombinant protein. No previous information was available on the clinically relevant epitopes on Cyn d 1. In the present study, we identified the immunodominant regions on the Cyn d 1 molecule. A collection of overlapping fragments and deletion mutants were expressed along with an N-terminal His tag and purified by Nickel affinity chromatography. The purified proteins were subjected to immuno dot blotting to compare their IgE reactivity with sera from BGP allergic patients. Fragments F1, F2 and F3, covering amino acid residues 1–120, failed to show IgE Ab binding for all sera tested. IgE binding to deletion mutants was weaker compared to intact rCyn d 1. We identified at least two major IgE-binding regions (120–170) and (224–244) by immunodot blot analysis. To further determine the binding of deletion mutants to IgE, a solid-phase ELISA was carried using four individual sera and serum pool from 10 patients with BGP allergy. Both deletion mutants D1 and D2 showed about 40% reduction in IgE binding. Interestingly, deleting 50 residues (120–170) reduced IgE binding to about the same extent as deleting the last 20 residues. The allergenic potency of D3 devoid of both epitopes produced a significant reduction of about 70% in IgE binding in comparison to the whole molecule. These findings clearly indicated that the C-terminal of Cyn d 1 was the major IgE-binding region. Since removal of the two immunodominant regions gave about 70% reduction in IgE reactivity of rCyn d 1, other minor epitopes may be present that account for the remaining 30% reactivity. The data derived from our study are in agreement with IgE epitopes described from other grass group 1 allergens. The involvement of C-terminal region in IgE binding has been reported for the group 1 pollen allergens from several grasses using either synthetic peptide fragments corresponding to linear segments of the allergen or monoclonal antibody based mapping strategies [20-22]. Tamborini and co-workers mapped the IgE binding region of Lol p 1, a major group 1 allergen from Rye grass pollen. They produced three deletion mutants with large deletions in the protein. The first mutant, lacking the N-terminus region (aa 1–88), showed 27% IgE binding, the second mutant, lacking region (aa 132–190), showed 6.3% IgE reactivity, and the third mutant, missing the C-terminus region (aa 116–240), showed 4.5% IgE reactivity as determined by ELISA. A strong reduction in IgE reactivity was measured for the third mutant suggesting the C-terminus as the immunodominant portion of Lol p 1 [25]. A similar approach using gene fragmentation and synthetic peptides was used to map the IgE-binding epitopes of major group 1 allergen of velvet grass pollen Hol l 1 [21] and Phl p 1 from timothy grass [22]. The binding patterns of patients' IgE to small peptides was much weaker than to larger fragments, and 46% of sera showed binding to C-terminus as opposed to only 8% of sera binding to N-terminus region in the case of the Hol l 1 molecule [21]. The findings from B-cell epitope mapping studies of Phl p 1 were also similar to Hol l 1, with the majority of sera recognizing the C-terminal fragment rT147 that comprises amino acids 146 through 240 of Phl p 1 [22]. The IgE-binding regions identified in Cyn d 1 in the current study partially or completely overlap with those identified in other Group 1 allergens where the C-terminal portion has been shown to be immunodominant. The contention that this IgE containing region is representative of group 1 allergens from other grass species, and therefore is relevant for grass pollen allergy in general, is supported by the fact that group 1 allergens show very high sequence similarity (~85%). In addition, this IgE containing regions has been reported to represent prominent IgE epitope bearing region of grass group 1 allergens from other grass species.

The entire population of IgE epitopes for a number of aeroallergens has been shown to be dependent on their structural integrity. One of the approaches used to reduce the anaphylactic potential of any allergen is to disrupt the overall secondary structure of the protein. Since the majority of IgE epitopes are known to be conformational, a collapse in the structure can prevent allergen-specific IgE recognition and cross-linking. In the extensively studied Bet v 1 allergen from birch pollen expressing the molecule as two separate fragments led to complete ablation of IgE reactivity [26]. In case of the fragments generated for Cyn d 1 in this study fragment 4 (aa 1–120), covering the amino-terminal half of the molecule, did not bind IgE while fragment 7 (aa 129–244), covering the C-terminal end, showed positive reaction with IgE. These results suggest that breaking the Cyn d 1 molecule into two halves did not have a significant effect on IgE reactivity. The finding that the majority of the IgE binding was preserved after removing 120 residues from the N-terminal provides further evidence for the C-terminus as a likely candidate representing the major IgE binding region on Cyn d 1.

Based on the findings from this study, the lack of cross reactivity of BGP extract with the Poeae grasses may seem puzzling since most of the IgE antibodies are directed against the C-terminal of the group 1 allergen across the different grasses, including Cyn d 1. One possible explanation for this phenomenon may be because several of the earlier studies were carried out with crude pollen extracts that comprised of a mixture of several allergenic proteins making it difficult to obtain a detailed IgE profile of the different allergenic fractions. Furthermore, three dimensional structure and steric orientation of allergens are crucial for cross-linking of mast cells and basophils-bound IgE antibodies for inducing release of inflammatory mediators. Spatial clustering of IgE-binding sites on the Phl p 1 surface has been shown to be an important factor for its allergenic activity. Results from this study defined a sterically oriented C-terminal fragment of Phl p 1 as the portion with highest allergenic activity [27]. Although group 1 allergens have high sequence identity, point mutations of very few key amino acid residues can drastically alter the protein structure and spatial orientation of the IgE epitope. For example, cysteine residues and disulfide bonds play an important role in IgE binding in the case of grass allergen Lol p 1. Replacement of cysteine residue at position 77 with serine caused an average of 67.2% reduction in human IgE reactivity to this molecule [28]. As discussed previously, even though the major IgE epitope for group 1 allergen is located at the C-terminus, there may be differences in the minor epitopes that could influence the secondary structure of the protein. Alterations of secondary structure may cause constraints on the orientation of the molecule. Small changes in amino acid sequence can alter a protein's structure that, in turn, can affect mast cell induction and basophil activation. Studies have demonstrated that isoforms of the same allergen with very few amino acid differences can exhibit greatly varying potencies to induce an allergic response. For example, a naturally occurring low allergenic isoform has been reported for hazel pollen allergen Cor a 1 [29]. Since the crystal structure of Cyn d 1 protein has not been solved, further studies are needed to finely map the IgE-binding regions of Cyn d 1. Inhibition of IgE binding by synthetic peptides representing homologous amino acids sequences of other Poaeae grasses could provide a better understanding of the precise distribution of IgE epitopes on Cyn d 1 and their role in allergic sensitization. This type of analysis should further elucidate the observed lack of cross reactivity between BGP and pollen from Poae grasses.

## Conclusion

In conclusion, we describe here the results of an experimental investigation that led us to identify at least two major IgE binding regions on Cyn d 1, the major allergen from Bermuda grass pollen. We mapped these binding regions to the C-terminal end of the molecule. The location of epitopes within the highly conserved C-terminus of grass group 1 allergens may be a common feature of the IgE response to grass group 1 allergens. Since structural and mutational analyses of allergens can be utilized to develop vaccines for allergy therapy, targeted substitution or deletion of amino acids within the identified epitopes could result in nonanaphylactic allergenic variants that may, in turn, be useful to develop vaccines for BGP allergy sufferers.

## Competing interests

The authors declare that they have no competing interests.

## Authors' contributions

RT carried out all the experimental studies and drafted the manuscript.

PLB revised the manuscript, provided intellectual content and general supervision.

MBS made substantial contribution in design and interpretation of data, drafting and revision of the manuscript for important intellectual content and general supervision.

All authors have read and approved the final manuscript.
